# 1071. Impact of volume resuscitation on heart rate variability in a model of hemorrhagic shock in pigs

**DOI:** 10.1186/2197-425X-2-S1-P87

**Published:** 2014-09-26

**Authors:** E Salomão, DA Otsuki, AL Corrêa, DT Fantoni, JOC Auler

**Affiliations:** Department of Surgery, Faculdade de Medicina da Universidade de São Paulo, São Paulo, Brazil; Department of Surgery, Faculdade de Medicina Veterinária e Zootecnia da Universidade de São Paulo, São Paulo, Brazil

## Introduction

Hemorrhagic shock is responsible for high mortality rates in civilian injuries and combat casualties. The initial care of these patients comprehends an early assessment of hypovolemia, bleeding management and fluid resuscitation [1], while an adequate autonomic function is essential for maintaining the hemodynamic stability during haemorrhage. The analysis of heart rate variability (HRV) has been shown as a promising noninvasive technique for assessing the cardiac autonomic modulation in trauma, and several recent studies have demonstrated an association between HRV and clinical outcome [2].

## Objectives

The objective of this study was to evaluate HRV during hemorrhagic shock and fluid resuscitation, comparing to traditional hemodynamic and metabolic parameters.

## Methods

Twenty pigs were submitted to acute hemorrhagic shock by removing 60% of the estimated blood volume in 15 minutes (3 mL/kg/min) and then these animals were evaluated for 60 minutes without any treatment. Surviving animals were treated with lactated Ringer solution (3:1) and evaluated for an additional period of 180 minutes. HRV metrics, as well as hemodynamic and metabolic parameters, were evaluated in survivors and non-survivors animals. Data were analysed using the repeated measures ANOVA or Friedman test (for normal and non-normal distributed date, respectively), followed by Tukey or Dunn's test as necessary. Surviving and non-surviving animals' data were compared by the unpaired t-test or Mann-Whitney U test. A multivariable logistic regression analysis was performed to estimate predictive factor for mortality resulting from hemorrhagic shock.

## Results

Seven of the 20 animals have died during haemorrhage and initial fluid resuscitation. All the animals presented an increase in the LF_nu_ and a decrease in the HF_nu_ values after haemorrhage. However, non-surviving animals presented lower LF_nu_ (72.2±18.6 versus 85.9±7.1) and higher HF_nu_ values (27.8±18.6 versus 14.1±7.1), as well as lower MAP and CI and higher levels of plasma lactate and potassium. Besides these results, the fluid resuscitation was not able to reinstate the LF and HF alterations, but restored hemodynamic and metabolic parameters.

## Conclusions

The HRV metrics were able to discriminate survivors from non-survivors only at late stages of hemorrhagic shock. Moreover, metabolic variables along with CI and MAP were more reliable to reflect hemorrhagic shock severity than HRV metrics.

## References

[1] Wilson M, Davis DP, Coimbra R (2003). Diagnosis and monitoring of hemorrhagic shock during the initial resuscitation of multiple trauma patients: a review. The Journal of Emergency Medicine.

[2] Winchell RJ, Hoyt DB (1996). Spectral analysis of heart rate variability in the ICU: a measure of autonomic function. The Journal of Surgical Research.

